# shp-2 gene knockout upregulates CAR-driven cytotoxicity of YT NK cells

**DOI:** 10.18699/VJ20.598

**Published:** 2020-02

**Authors:** V.G. Subrakova, S.V. Kulemzin, T.N. Belovezhets, A.N. Chikaev, N.A. Chikaev, O.A. Koval, A.A. Gorchakov, A.V. Taranin

**Affiliations:** Institute of Molecular and Cellular Biology of Siberian Branch of the Russian Academy of Sciences, Novosibirsk, Russia Novosibirsk State University, Novosibirsk, Russia; Institute of Molecular and Cellular Biology of Siberian Branch of the Russian Academy of Sciences, Novosibirsk, Russia; Institute of Molecular and Cellular Biology of Siberian Branch of the Russian Academy of Sciences, Novosibirsk, Russia Novosibirsk State University, Novosibirsk, Russia; Institute of Molecular and Cellular Biology of Siberian Branch of the Russian Academy of Sciences, Novosibirsk, Russia; Institute of Molecular and Cellular Biology of Siberian Branch of the Russian Academy of Sciences, Novosibirsk, Russia; Institute of Molecular and Cellular Biology of Siberian Branch of the Russian Academy of Sciences, Novosibirsk, Russia Institute of Chemical Biology and Fundamental Medicine of Siberian Branch of the Russian Academy of Sciences, Novosibirsk, Russia; Institute of Molecular and Cellular Biology of Siberian Branch of the Russian Academy of Sciences, Novosibirsk, Russia Novosibirsk State University, Novosibirsk, Russia; Institute of Molecular and Cellular Biology of Siberian Branch of the Russian Academy of Sciences, Novosibirsk, Russia Novosibirsk State University, Novosibirsk, Russia

**Keywords:** NK cells, CRISPR/Cas9, CAR-NK, Shp-2, NK-клетки, CRISPR/Cas9, CAR-NK, Shp-2

## Abstract

In Russia, cancer is the second leading cause of death following cardiovascular diseases. Adoptive transfer of NK cells is a promising approach to fight cancer; however, for their successful use in cancer treatment, it is necessary to ensure their robust accumulation at tumor foci, provide resistance to the immunosuppressive tumor microenvironment, and to engineer them with higher cytotoxic activity. NK lymphocytes are known to kill cancer cells expressing a number of stress ligands; and the balance of signals from inhibitory and activating receptors on the surface of the NK cell determines whether a cytotoxic reaction is triggered. We hypothesized that stronger cytotoxicity of NK cells could be achieved via gene editing aimed at enhancing the activating signaling cascades and/or weakening the inhibitory ones, thereby shifting the balance of signals towards NK cell activation and target cell lysis. Here, we took advantage of the CRISPR/Cas9 system to introduce mutations in the coding sequence of the shp-2 (PTPN11) gene encoding the signaling molecule of inhibitory pathways in NK cells. These shp-2 knock-out NK cells were additionally transduced to express a chimeric antigen receptor (CAR) that selectively recognized the antigen of interest on the target cell surface and generated an activating signal. We demonstrate that the combination of shp-2 gene knockout and CAR expression increases the cytotoxicity of effector NK-like YT cells against human prostate cancer cell line Du-145 with ectopic expression of PSMA protein, which is specifically targeted by the CAR.

## Abstract (ru)

Одна из самых распространенных причин смертей пациентов в России, наряду с болезнями системы кровообращения, – это онкологические заболевания. Перспективным средством в борьбе с раковыми клетками представляются NK-клетки (естественные киллеры), однако для успешного применения в терапии онкологических заболеваний необходимо обеспечить их накопление в опухолевых очагах, устойчивость к иммуносупрессивному микроокружению, а также более высокую цитотоксическую активность. Известно, что NK-лимфоциты уничтожают раковые клетки, экспрессирующие специфические стресс-лиганды; при этом баланс сигналов от ингибирующих и активирующих рецепторов на поверхности NK-клетки определяет, будет ли запущена цитотоксическая реакция. Один из теоретически возможных способов повышения цитотоксичности состоит в том, чтобы при помощи генетического редактирования усилить активационные сигнальные каскады в NK-клетках и/ или ослабить ингибирующие, таким образом сместив баланс сигналов в сторону активации лимфоцитов и лизиса мишеней. NK-клетки с таким модифицированным цитотоксическим потенциалом могут эффективнее уничтожать раковые мишени, обладающие так называемой устойчивостью к лизису. В этой работе мы предлагаем дважды модифицировать NK-клетки. Во-первых, при помощи системы CRISPR/Cas9 проводить нокаутирование гена shp- 2 (PTPN11), кодирующего белок Shp-2 – негативный регулятор активации NK-клеток; во-вторых, при помощи лентивирусных векторов интегрировать кассету, кодирующую CAR (химерный антигенный рецептор), способный специфично связываться с антигенами на поверхности раковой мишени и генерировать активирующий сигнал. В качестве модельной NK-клеточной линии нами была выбрана перевиваемая линия NK-подобного фенотипа YT, поскольку эти клетки не нуждаются в специфических цитокинах для культивирования и могут проявлять перфорин/гранзим-опосредованную цитотоксическую активность. Мы показали, что сочетание нокаута гена shp-2 и экспрессии CAR повышает ци- тотоксичность эффекторных клеток на модели NK-устойчивой клеточной линии аденокарциномы простаты человека Du-145, экспрессирующей специфично узнаваемый CAR антиген, белок PSMA. Подобные линии с «усиленным» цитотоксическим фенотипом в перспективе могут быть использованы для нужд противорако- вой терапии.

## Introduction

Natural killer cells are cells whose primary function is to eliminate infected and transformed cells in the body. Cytotoxic activity of NK cells is regulated by the balance of signals triggered by the interaction of activating and inhibitory receptors with appropriate ligands on the surface of target cells (Malarkannan, 2006; Lee, Gasser, 2010; Becker et al., 2016; Del Zotto et al., 2017).

Inhibitory receptors play an central role in NK cell biology since most of their ligands are represented by Major Histocompatibility Complex I (MHC-I) molecules, that are present on the surface of nearly all healthy cells and frequently absent on cancer or infected cells (Hewitt, 2003; de Charette et al., 2016). This ensures that normal cells are spared by NK cells, whereas the cells that have lost MHC-I expression become eliminated (Kärre et al., 1986; Hanke et al., 1999). Nevertheless, absence of MHC-I expression does not universally guarantee that an NK cell would kill a target cell, as this activity is also heavily dependent on the presence of ligands to activating receptors of NK cells (Cerwenka et al., 2001; Paul, Lal, 2017). Inhibitory receptor signal is known to be mainly transduced via SH2-containing inositol 5′ polyphosphatase 1 (SHIP-1) and/or tyrosine phosphatases Shp-1 and Shp-2. The latter two proteins dephosphorylate tyrosine residues within the ITAM motifs of activating receptors thereby aborting the signaling cascade (Rehman et al., 2018).

NK cells are successfully used in therapy of oncological diseases (Rezvani et al., 2017). Nevertheless, because tumor cells actively withstand elimination by establishing an immunosuppressive tumor microenvironment, the cytotoxicity of NK cells can be strongly affected (Mamessier et al., 2011; Pasero et al., 2016; Suen et al., 2018). There are several ways of enhancing the cytotoxicity of NK cells. These include modification of signaling pathways, altering the levels and repertoire of NK cell receptors, or boosting the activity of NK cells with appropriate cytokines (Igarashi et al., 2004; Childs, Carlsten, 2015; Yang et al., 2017; Freund-Brown et al., 2018; Nayyar et al., 2019). In this study, we explored whether modification of the inhibitory signaling pathway through the shp-2 gene knockout in NK cells would result in the increase of the cytotoxic activity of model NK cells.

## Materials and methods

**Cell lines and cell culture.** YT, Du-145-PSMA, and HEK293T cell lines were maintained in Iscove’s Modified Dulbecco’s Medium (IMDM) supplemented with 10 % fetal bovine serum (FBS), 100 U/ml penicillin, and 100 μg/ml streptomycin (Sigma) in a humidified atmosphere of 5 % CO2 at 37 °C.

**Plasmid construction.** Two shp-2-specific sgRNAs were identified using publicly available bioinformatics tools (Moreno- Mateos et al., 2015; Doench et al., 2016). The following target sequences were selected: gtgcagatcctacctctgaaagg and acagtactacaactcaagcagg (PAM site underlined).

The lentiCRISPRv2 vector provided by Prof. Feng Zhang (Addgene # 52961, USA) (Sanjana et al., 2014) was used for co-delivery of sgRNAs and Cas9. Oligonucleotides corresponding to the selected targets were denatured in a boiling water bath followed by slow renaturation and cloned into the lentiCRISPRv2 vector between BsmBI restriction sites.

The plasmid DNA of clones lentiCRISPRv2-Shp2g1 and lentiCRISPRv2-Shp2g2 encoding shp2-specific sgRNA1 and sgRNA2, was mixed with the DNA of packaging plasmids psPAX2 and pMD2.G (provided by Prof. D. Trono) in the following weight ratio: 10:10:7.5:2.5, in the total amount of 3 μg. Using calcium phosphate transfection (Kutner et al., 2009), the resulting plasmid DNA mix was delivered to HEK293T cells. Supernatants containing VSV-G pseudotyped lentiviral particles were collected 48 hours after transfection, filtered through 0.45 μm PES filters and used either fresh or stored at –70 °C.

**YT cell transduction.** To improve transduction rate of YT cells, a spinoculation protocol was used (O’Doherty et al., 2000). YT cells were seeded in 24-well plates (1 × 105 cells) in the presence of polybrene (8 μg/ml) followed by the addition of supernatants containing pseudotyped lentiviral particles. Cells were centrifuged at 500 g for 40 minutes at 32 °C and incubated in a CO2 incubator for 16 hours. The next day, the supernatant was replaced with a fresh culture medium. After 3 days, selection of transduced cells was performed by adding puromycin (Invitrogen, USA) to a final concentration of 5 mg/ ml (1 week), with non-transduced cells serving as controls.

**Western blot analysis.** For western blot analysis, transduced YT cells and control non-transduced cell lines YT-wt and HEK293T were lysed in lysis buffer (100 mM Tris, pH 6.8, 2 % SDS, 5 % β-mercaptoethanol, 15 % glycerol). Cell lysates were centrifuged, boiled in a water bath for 5 minutes, separated by SDS-PAGE in a 10 % polyacrylamide gel and transferred onto a nitrocellulose membrane (GE Healthcare, USA). After blocking in 3 % milk in PBST, the membrane was incubated with rabbit monoclonal antibodies against Shp-2 (1 : 3000, # 3397S, Cell Signaling Technology, USA), followed by an HRP-labeled secondary goat-anti-rabbit antibody (1:8000, produced in-house). Equal loading was controlled by using hybridization with control antibodies against β-actin (1:3000, # ab3280, Abcam, USA). Signal was detected using an ECL-prime substrate in accordance with the manufacturer’s recommendations (GE Healthcare, USA) and Amersham Imager 600 with an exposure time of 1 min.

**Flow cytometry.** For phenotyping CAR-YT^shp-2–/–^ cell lines, 105 cells were washed in PBS and incubated for 30 minutes with the biotinylated protein L (3.3 μg/ml) (M00097, Genscript, USA) at a temperature of 4 °C. Then the cells were washed and incubated with streptavidin-APC (Thermo Fisher, USA) in accordance with the manufacturer’s recommendations. Vital dye 7AAD (Biolegend, USA) was used to exclude dead cells from the analysis. The samples were analyzed using BD FACSCanto® II (Becton Dickinson and Company) and BD FACSDiva software.

**Real Time Cytotoxicity Assay (RTCA).** Adherent target cells (5 × 104 cells per well) were seeded in 8-well E-plates (ACEA Biosciences, Korea) and left to grow for 16–18 hours. The next day, the medium was removed and replaced with a fresh one containing 105 effector cells. Cell growth was monitored for 24 hours using the RTCA iCELLigence system. Cytotoxicity was calculated using the formula: [CI (target cells without effector cells) – CI (target cells with effector cells) × 100/CI (target cells without effector cells)], where CI is the normalized impedance value in the wells (Golubovskaya et al., 2017).

**Flow cytometry-based cytotoxicity assay.** Cells were labeled with Cell Proliferation Dye eFluor 670 (Thermo Fisher, USA). 50,000 target cells per well were seeded into a 96-well plate. Next, an equal number of effectors was added (target cells without effectors in several wells were left as a control). Cells were incubated for 4 hours in a humidified atmosphere of 5 % CO2 at 37 °C. Then the vital dye 7AAD was added to each well and the percentage of living target cells was measured on a BD FACSCantoII flow cytometer.

**Statistical analysis ** was performed using Prism software (GraphPad version 8.0). One-way analysis of variance (oneway ANOVA) was used to detect the differences between the activity of control cell lines that did not carry the shp-2 gene knockout and that of knockout cells. All data are presented as mean ± standard error of the mean.

## Results

**Fig. 1.**
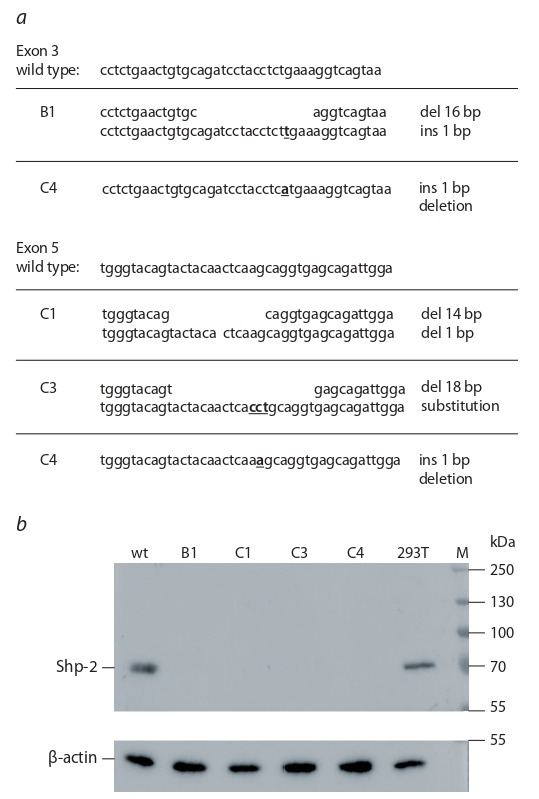
Verification of shp-2 knock-out in the monoclonal YT cell line derivatives. a, nucleotide sequences of CRISPR/Cas9-induced mutations in the exons 3 and 5 of the shp-2 gene; b, western-blot hybridization of cell lysates prepared from shp-2-mutant cell lines B1, C1, C3, and C4, as well as from the control HEK293T (293T) and YT (parental cell line, wt) cells with Shp-2-specific or betaactin (loading control) antibodies.

In this study, we have chosen human immortalized cell line YT that has an NK-like phenotype. These cells are known to display perforin-mediated target cell lysis, lack Fc receptor expression and do not require conditioning of growth media with IL2 (Yodoi et al., 1985; Deaglio et al., 2002; Edsparr et al., 2010). To knock-out shp-2, CRISPR/Cas9 system was used. Two sgRNAs specific to the 3rd and 5th exons of the gene were cloned into a lentiviral vector lentiCRISPRv2 (Sanjana et al., 2014). The two resulting constructs lentiCRISPRv2- Shp2g1 and lentiCRISPRv2-Shp2g2 were used for producing VSV-G-pseudotyped lentiviral particles, which then were used for transduction of YT-cells. Given that lentiCRISPRv2 vector carries a puromycin resistance gene, transduced cells were subjected to antibiotic selection and single-cell cloning. A panel of monoclonal derivatives of YT cells was analyzed by Sanger-sequencing the genomic fragments centered at sgRNA-binding sites in the shp-2 gene, in order to identify the clones carrying biallelic mutations in this gene (Fig. 1, a).

Four clones (B1, C1, C3, and C4) were obtained that displayed truncated shp-2 sequence (see Fig. 1, a). Knock-outs were verified by western-blot analysis, which confirmed that these clones did not express a full-length Shp-2 protein (see Fig. 1, b).

**Fig. 2.**
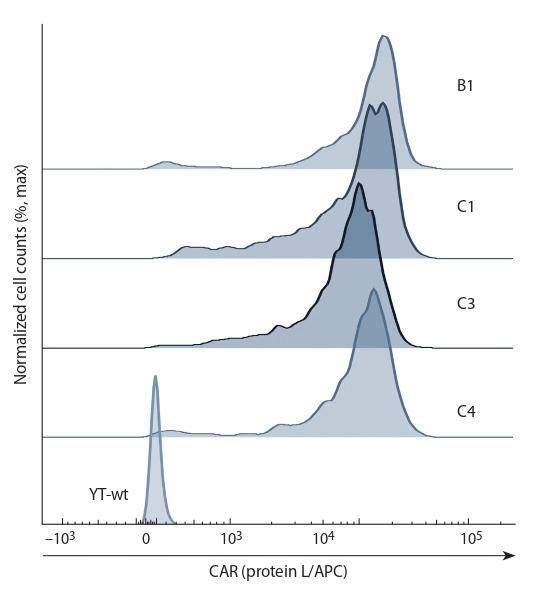
Cell surface expression of CAR in shp-2-knockout CAR-YT cells (B1, C1, C3, C4) vs control parental YT cells (YT-wt).

One way to re-target NK cell cytotoxicity is via expression of chimeric antigen receptors (CARs). Even if the target cell lacks typical stress markers recognized by endogenous NK cell receptors, binding of a CAR to its cognate target may induce pro-activation signaling in a CAR-NK cell. To test whether CAR-mediated cytotoxicity is enhanced upon shp2-knock out, we used our previously published second-generation CAR (scFv(J591)-CD8hinge-CD28TM-CD28-CD3z) (Kulemzin et al., 2019) that is specific for PSMA, a common surface marker of prostate cancer cells (Chang, 2004; Gorchakov et al., 2019). Wild-type and shp2-mutant YT cell lines were transduced to express this CAR, which was verified by flow cytometry (Fig. 2).

**Fig. 3.**
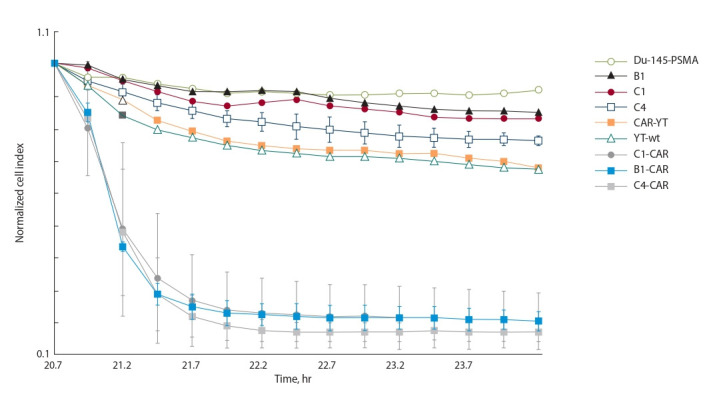
RTCA of cell cytotoxicity of shp-2-knockout cell lines B1, C1, and C4, CAR-expressing derivatives of B1, C1, and C4, as well as of control CAR-YT and YT-wt cell lines against target Du-145-PSMA cells. Shown is the normalized cell index following the addition of effector cells.

Next, we compared the cytotoxic activity of the B1-CAR, C1-CAR, and C4-CAR cells vs CAR-YT cells against Du- 145 prostate cancer cells engineered to ectopically express PSMA. Du-145 cells have been reported to be resistant to NK cell killing (Hood et al., 2019), which establishes them an appropriate model for assaying possible enhancement of NK cell-mediated cytotoxicity. We used iCELLigence platform (ACEA Biosciences, Korea) for the cytotoxicity analysis. It was found that CAR-YT^shp-2–/–^ cells exerted significantly higher cytotoxicity than CAR-YT cells with a functional shp- 2 gene; no difference between the knock-out subclones was observed (Fig. 3).

**Fig. 4.**
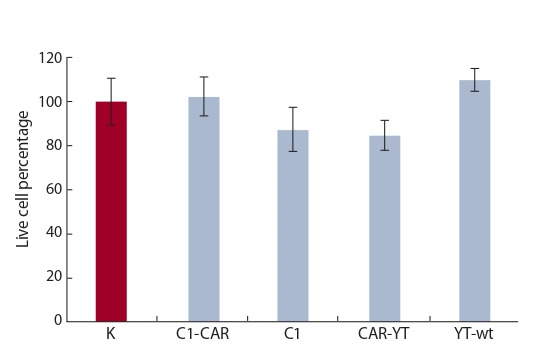
Analysis of cytotoxic activity of YT cells mutant for shp-2 (C1-CAR, C1) or harboring wild-type shp-2 gene (CAR-YT, YT), against peripheral blood mononuclear cells of a healthy donor. Incubation time was 4 hrs at a 1:1 E:T ratio. K – no effector cells were added. Average and standard deviation values for at least three independent measurements are shown.

To understand whether CAR-YT^shp-2–/–^ cells may display unwanted cytotoxicity against healthy cells, cytotoxicity assay was conducted with PBMCs isolated from a healthy donor. We observed that the percentage of dead normal cells did not differ significantly between the wells where CAR-YT^shp-2–/–^ cells or no effector cells (control) were added (Fig. 4). This was also true for cultures, where normal cells were co-incubated with either CAR-YT or CAR-YT^shp-2–/–^ cells.

## Discussion

Cytotoxic activity of NK cells is known to be regulated by the balance of activating and inhibitory receptor signals (Lee, Gasser, 2010; Sivori et al., 2019). Cytotoxic reaction occurs when two conditions are met. Namely, if an activating signal is present, (i. e. when the target cell expresses the ligand(s) to the activating receptors) and if the negative signaling is sufficiently reduced or absent (i. e. the cell lacks expression of the ligands of inhibitory NK cell receptors, or conversely, an NK cell lacks an inhibitory receptor to the ligand) (Chester et al., 2015; Pasero et al., 2016). Whenever positive signaling is absent or negative signaling outweighing the positive signaling, cytotoxic reaction will not be launched. In the context of NK cell therapy, this translates to the failure to eliminate the tumor cells (Pasero et al., 2016; Del Zotto et al., 2017).

Absence of activating signal can be effectively solved by engineering CAR expression in NK cells, an approach already actively used in practice (Imai et al., 2005; Rusakiewicz et al., 2013; Quintarelli et al., 2018; Ingegnere et al., 2019). Nonetheless, tumor cells overexpressing the ligands of inhibitory NK cell receptors may still remain protected from CAR-NK cell mediated lysis (Rezvani et al., 2017).

One way to address this problem is to create NK cells with knock-outs of inhibitory receptors. Although technically feasible, this may not be as straightforward, considering the large number of these receptors. In our opinion, knocking out Shp-2, a single “hub” of NK cell signaling appears as a viable alternative (Yusa, Campbell, 2003; Purdy, Campbell, 2009). Our experiments show that YT cell line derivatives lacking SHP2 expression display stronger CAR-mediated cytotoxicity towards NK-resistant cell line Du-145-PSMA (Hood et al., 2019), unlike CAR-YT cells (see Fig. 3). This is likely attributable to the fact that Du-145-PSMA cells have a high density of inhibitory ligands, which results in the suppression of activating signaling in NK cells, even upon CAR engagement. Indeed, it was reported that Du-154 cells express low levels of ligands to the activating NK receptors NKp30 and NKp46, while MHC-I expression is very high (Pasero et al., 2015).

Safety of such combination is yet to be found, as it is possible that this may lead to increased cytotoxicity towards healthy tissues, as a result of weakening of inhibitory signaling. To ensure that the YT^shp-2–/–^ cells are safe, testing on a wide panel of different healthy cell types obtained from multiple donors may be required, which is a challenging task from a technical and ethical points of view. In the current study, we have measured cytotoxicity of CAR-YTshp-2–/– cells towards the lymphocytes of a healthy donor, and no significant difference with negative control was observed. This suggests that reducing the inhibitory signaling in NK cells likely poses no threat provided that no strong activating signals are available. However, to address this important safety concern a more comprehensive study is indeed required. We envisage that a representative and diverse panel iPS-derived normal human cell types can be obtained to test for the possible off-target activity of shp2-mutant YT cells.

## Conclusion

Thus, the best option for enhancing cytotoxicity of NK cells and the YT cell line in particular is to induce activating signals using CARs in combination with the suppression of inhibitory signaling via knocking out its key mediator, the SHP-2 protein. In the future it is necessary to comprehensively study the safety of modified lymphocytes, in particular, to study their cytotoxic activity against a wider range of healthy tissues.

## Conflict of interest

The authors declare no conflict of interest.
